# A case report of secondary parathyroid adenomatous hyperplasia with carcinoma

**DOI:** 10.1097/MD.0000000000031362

**Published:** 2022-11-18

**Authors:** Simei Chen, Xin Sui, Bingxin Zhao, Zongjie Liu, Xinpeng Dai, Yang Ding

**Affiliations:** a Department of Ultrasound Medicine, The Thrid Hospital of Hebei Medical University, Shijiazhuang Hebei Province, PR China; b Department of pathology, The Thrid Hospital of Hebei Medical University, Shijiazhuang Hebei Province, PR China.

**Keywords:** parathyroid, secondary parathyroid hyperplasia cancer, ultrasonography

## Abstract

Secondary parathyroid hyperplasia canceration is very rare and thus easily be overlooked during parathyroid ultrasound examination. However, secondary parathyroid hyperplasia still has the possibility of canceration, and it is still important to be alert to its occurrence when performing ultrasound examinations and clinical treatment.

**Patient concerns:**

A 49-years-old man visited our outpatient department with generalized weakness and pain in both lower extremities a month ago.

**Diagnosis:**

Hyperparathyroidism secondary to chronic renal failure.

**Interventions:**

The patient underwent ultrasound and other preoperative examinations. The preoperative ultrasound showed 3 parathyroid enlargements, 2 on the left and 1 on the right. The patient then underwent surgical treatment.

**Outcomes:**

Ultrasonography suggested the presence of 3 parathyroid hyperplasias, and ectopic right inferior parathyroid gland was visible during intraoperative examination. 10 days after surgery, the patient’s Parathyroid Hormone returned to the normal range.

**Conclusion:**

Secondary parathyroid hyperplasia has the potential to become cancerous, so doctors should be alert to its occurrence when conducting ultrasound examinations. Ultrasound examination is the key to its diagnosis and subsequent treatment.

## 1. Introduction

Because ultrasound is highly sensitive, specific, economical, and convenient, it has become the preferred modality for examining the parathyroid glands. Especially in hyperplastic parathyroid glands, ultrasonography can show the number, size, internal echogenicity, and blood flow changes of the parathyroid glands and the adjacent borders, and also has good visualization of the echogenic features around the lesion and calcifications in the parathyroid glands. Although there are still some cases of misdiagnosis and underdiagnosis, ultrasonography still has considerable advantages for ectopic parathyroid glands in the thyroid gland. Ultrasonography can clearly show the blood flow and distribution in the thyroid gland, which makes it easier to diagnose the type of disease when compared to the pattern of blood flow in patients with clear parathyroid enlargement.^[1]^ For other ectopic secondary parathyroid glands, multiple imaging examinations can be combined for preoperative localization, thus shortening the operative time and reducing surgical injuries and postoperative complications.

## 2. Case presentation

**Figure 1.**
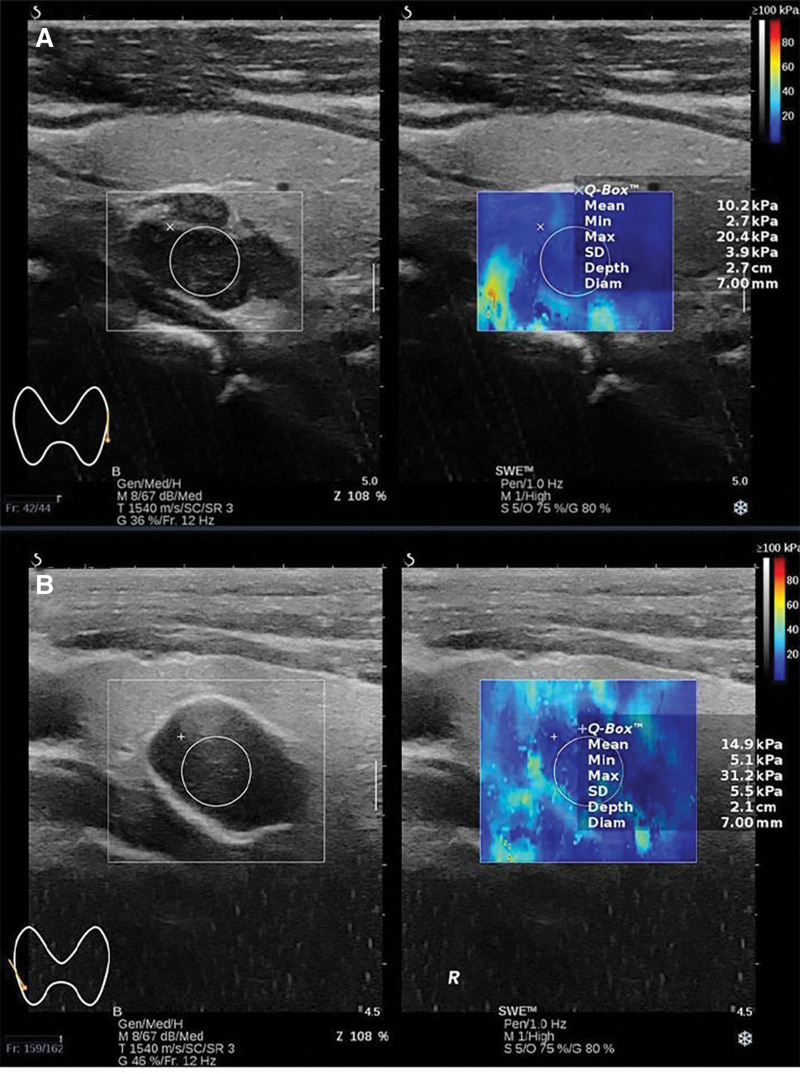
(A) Shear-wave ultrasound imaging of secondary hyperparathyroidism. (B) Shear-wave ultrasound imaging of secondary parathyroid hyperplasia. It is obvious that the Young’s modulus value of secondary parathyroid carcinoma is significantly higher than that of secondary hyperparathyroidism.

**Figure 2.**
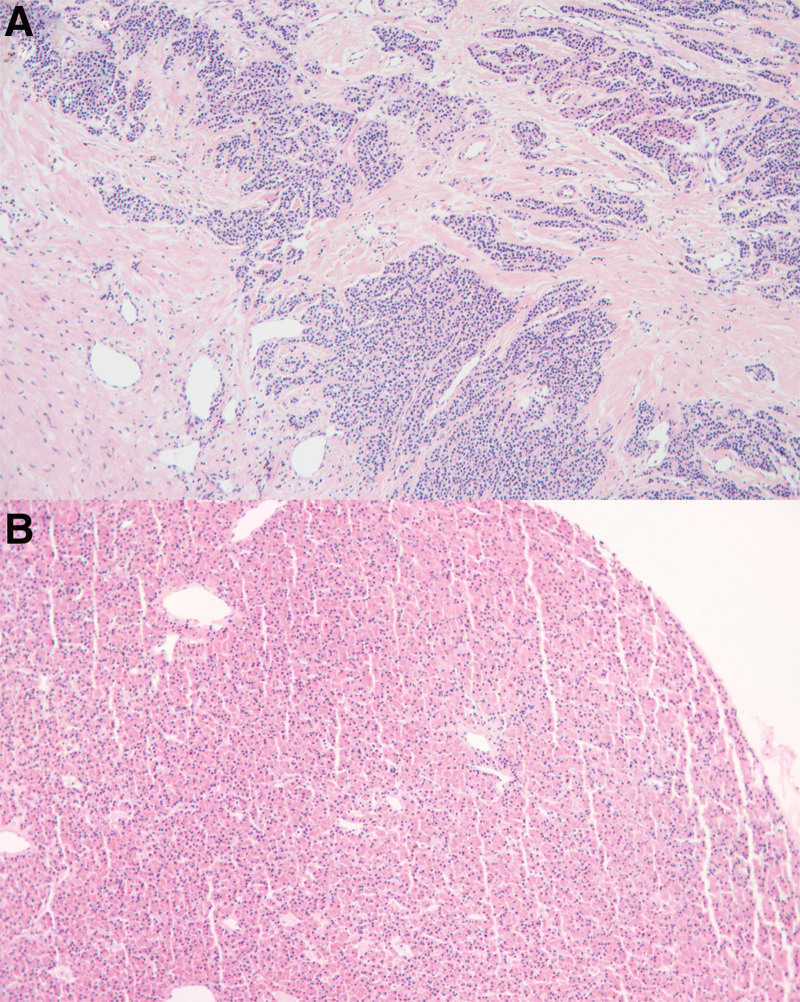
(A) Pathological image of secondary parathyroid carcinoma: Tumor cells infiltrate the surrounding soft tissue, arranged in sheet and beam shape, with light staining and uniform nuclei. (hematoxylineosin [H&E], original magnification 100). (B) Pathological picture of secondary parathyroid hyperplasia: Diffuse hyperplasia of parathyroid chief cells. (hematoxylineosin [H&E], original magnification 100).

The patient, male, 49 years old, underwent related examinations due to generalized weakness and pain in both lower extremities a month ago, Parathyroid Hormone (PTH): 3000pg/mL, had a history of chronic renal failure for more than 10 years, and was diagnosed as secondary hyperparathyroidism. For further diagnosis and treatment, he visited our outpatient department and was scheduled for surgery. Ultrasound examination of the parathyroid glands: multiple hypoechoic nodules were seen in the bilateral parathyroid area. Two of them can be seen on the left side, which are located in the middle and dorsal side of the left lobe of the thyroid gland and below the lower pole of the left lobe of the thyroid gland. Larger nodule about 1. 4 × 0. 8cm in size with still clear borders and rich blood flow signal. One is visible on the right side, located in the right upper middle lobe of the thyroid posteriorly, with a size of about 1. 8 × 1. 2cm, still clear borders, rich blood flow signal, and a strong echogenic ring around it. We considered parathyroid hyperplasia. Parathyroid shear wave elastic modulus examination: left upper parathyroid Young’s modulus value Mean: 8. 4Kpa, left lower parathyroid Young’s modulus value Mean: 3. 1Kpa, right upper parathyroid Young’s modulus value Mean: 14. 9Kpa. Laboratory examinations after admission: PTH: 1483. 10pg/ml, CA: 2. 59mmol/L, P: 1. 92mmol/L. Parathyroid dual-phase imaging: An area of elevated radioactivity equivalent to the upper pole of both lobes of the thyroid gland and below, considered to be hyperfunctioning parathyroid tissue. The patient met the surgical indications and no contraindications to surgery were found in the preoperative examinations. The patient and his family were informed of his condition and agreed to undergo parathyroidectomy and autologous transplantation. Combining all the examinations, the surgeon considered that the right inferior parathyroid gland was ectopically located behind the sternum and decided to perform an additional posterior median sternotomy. Intraoperative: 3 parathyroid glands suggested by ultrasonography, all of which were confirmed by pathology as parathyroid tissue. Ectopic parathyroid glands located above the brachiocephalic trunk, medial to the internal jugular vein (Fig. 1). Postoperative pathological examination: both upper left and lower left parathyroid glands were hyperplastic. Right upper parathyroid adenomatous hyperplasia with calcification, seeing envelope invasion, was consistent with adenomatous hyperplasia carcinoma of the parathyroid. Immunohistochemical results: P53 (wild +), CgA (+), Cyclin D1 (+), Ki-67 (+, <5%), CD31 (-), PTH (+). Right inferior parathyroid adenomatous hyperplasia with calcification, carcinoma. Immunohistochemical results: D2-40 (-), Cyclin D1 (+), CgA (+), Ki-67 (+, <5%), CD34 (-), CD31 (-), PTH (+) (Fig. 2). 10 days after surgery, the patient’s blood calcium was controlled at 1.80-2.20 mmol/L and PTH returned to the normal range.

## 3. Discussion

Parathyroid carcinoma is a rare endocrine tumor in my country.^[2]^ Early detection and early surgical treatment can effectively correct calcium and phosphorus metabolism disorders. Parathyroid cancer has a low incidence, usually accounting for < 1% of primary hyperparathyroidism,^[3,4]^ but has been reported in Asian population as high as 8.1%.^[5]^ As a rare endocrine tumor, parathyroid cancer is highly malignant and needs to be highly alerted to its occurrence, while secondary parathyroid cancer is even rarer and rarely reported at home and abroad. The probability of secondary hyperparathyroidism being cancerous is extremely low, but it is still possible to become cancerous. In the future when patients with chronic renal failure undergo ultrasound examination for secondary hyperparathyroidism, doctors should always pay attention to the occurrence of cancer and should not ignore ultrasound diagnosis because of its rarity. Two-dimensional ultrasound manifestations of hyperparathyroidism secondary to maintenance hemodialysis: The hyperplastic parathyroid glands are enlarged, with clearly discernible glandular borders, round or oval, with hypoechoic internal echogenicity, some of which are inhomogeneous and nodular in shape, and some of which have foci of calcification within the hypoechogenicity.^[6]^ The main ultrasound signs of primary parathyroid carcinoma are large tumor (>3 cm), irregular margins, aspect ratio > 1, inhomogeneous echogenicity, calcification, and local tissue infiltration.^[7]^ This case is a secondary parathyroid carcinoma, and its ultrasonographic appearance is large, and the interior is hypoechoic and heterogeneous. This is almost the same as the ultrasound manifestations of the above 2 diseases. The secondary parathyroid carcinoma in this article has annular calcifications, while other hyperplastic parathyroid glands have interior calcifications without complete annular calcifications around them. When patients with secondary hyperparathyroidism show annular calcification on ultrasound, the possibility of secondary parathyroid cancer is increased. However, now that the data are single and lack of large sample support, we need to be especially alert to the occurrence of cancer when performing secondary parathyroid screening in the future to avoid missed and misdiagnosis. We performed ultrasound shear wave elastic modulus values based on parathyroid ultrasonography and found that Young’s modulus values were significantly higher in secondary parathyroid carcinoma than in other hyperplastic parathyroid glands. Some scholars^[8]^ performed routine ultrasound and shear wave elastography on parathyroid glands in patients with secondary hyperparathyroidism and found that parathyroid lesions with different levels of intact parathyroid hormone (iPTH) in the whole segment had different imaging features. Patients were divided into 4 groups according to their iPTH. As the iPTH gradually increased, the mean, maximum, and standard deviation of the Young’s modulus values of shear elastography showed an increasing trend, but the Young’s modulus values of other groups were staggered and did not strictly conform to the rule. In the present case, the same phenomenon existed: the Young’s modulus value of parathyroid glands with tumor-like hyperplasia and canceration is significantly higher than that of other non-cancerous hyperplasia parathyroid glands, but the sample size is small, there are random factors or human errors, and the results still need to be more sample size data to support. In the ultrasound examination of patients with secondary hyperparathyroidism, we cannot ignore the possibility of parathyroid cancer. Because the hardness of conventional cancerous lesions is > that of benign lesions when secondary parathyroid hyperplasia is found to have annular calcification and increased shear wave elastic modulus values, it suggests that secondary parathyroid cancer may be. At this time, the ultrasound department needs to prompt the surgeon to take a reasonable surgical plan to avoid missed diagnoses and misdiagnosis.

The overall incidence of ectopic parathyroid gland in the human body is high.^[9]^ Lower pole parathyroid ectopic is more common.^[10]^ During embryogenesis, the inferior parathyroid gland may migrate to different anatomical sites, such as the mediastinum, carotid sheath, thymus, and thyroid.^[11]^ Ectopic parathyroid brings many difficulties to clinical diagnosis and treatment. The number of secondary parathyroids is generally 3 to 7, and the most common number (above 80%) is 4.^[12,13]^ In this case, during the ultrasonography of the parathyroid glands, only 3 parathyroid glands were found in conventional locations, which were located on the back of the left and right lobes of the thyroid. The right inferior parathyroid gland was found at the root of the neck, and the right inferior parathyroid gland was considered to be possibly ectopic. In the thyroid, the carotid sheath, the upper mediastinum, and other common parathyroid ectopic locations, the right lower parathyroid is not found, and it is considered that the right lower parathyroid has a high probability of being ectopic behind the sternum. The ultrasound department recommended adding a parathyroid duplex examination. The results of the parathyroid duplex examination showed an area of increased radioactivity in the upper and lower parts of both lobes of the thyroid gland, which was considered to be hyperfunctional parathyroid tissue. The ultrasound department also recommended exploring the presence of parathyroid glands behind the sternum at the time of surgery. The surgery combined parathyroid ultrasonography, parathyroid duplex examination, and clinical situation, and decided to perform an additional posterior median sternotomy, which revealed that the right inferior parathyroid gland was located above the head and arm trunk and medial to the internal jugular vein. This ectopic hyperplastic parathyroid gland was removed during surgery, and the PTH returned to the normal range after surgery, suggesting that all parathyroid glands were removed. Preoperative parathyroid ultrasonography provided a good indication for surgical exploration, and the surgeons improved the surgical plan in time to avoid secondary surgery and intraoperative patient injuries, such as severe bleeding or retropharyngeal nerve dysfunction, and to improve patient prognosis.^[14]^ It can be seen that when the number of secondary parathyroid hyperplasia is uncertain, and only 1 to 3 parathyroid glands are found in conventional locations, it is highly suggestive that the parathyroid glands may be ectopic. Since routine parathyroid scanning alone is not sufficient to meet clinical needs, additional scanning of common ectopic locations of the thyroid should be performed to increase the detection rate of the parathyroid glands and thus increase the success rate of surgery. The histopathology of this case showed: adenomatous hyperplasia and carcinomatosis with calcification in the upper and lower right parathyroid glands, and capsular invasion was seen. According to World Health Organization criteria, the diagnosis of parathyroid carcinoma requires definitive lymphovascular or perineural invasion, or invasion into adjacent structures, or metastatic disease. The characteristic pathology of parathyroid carcinoma shows a supracellular tumor with trabecular growth, a thick fibrous band, and a thick fibrous tumor envelope. Other features include tumor necrosis, spindle-shaped tumor cells, prominent large nucleoli, and atypical nuclear schizophrenia.^[15]^

## 4. Conclusion

Secondary parathyroid cancer is very rare in China and Abroad, and it is easily missed and misdiagnosed by conventional ultrasonography, resulting in untimely treatment and wrong choice of surgical treatment. Secondary parathyroid hyperplasia has the potential to become cancerous and we need to be alert to its occurrence during ultrasonography. Traditional ultrasonic examination combined with shear wave elastic modulus measurement can improve the detection rate of secondary parathyroid carcinoma.

## Acknowledgements

We are grateful for the support given.

## Author contributions

**Conceptualization:** Sui Xin, Chen Simei.

**Data curation:** Chen Simei, Zhao Bingxin.

**Formal analysis:** Chen Simei, Liu Zongjie.

**Funding acquisition:** Chen Simei, Sui Xin.

**Investigation:** Chen Simei.

**Resources:** Chen Simei, Ding Yang.

**Project administration:** Chen Simei, Sui Xin.

**Supervision:** Chen Simei, Sui Xin.

**Writing ‐ original draft:** Chen Simei.

**Writing ‐ review & editing:** Chen Simei, Sui Xin.
